# Case of Periodical Spasm Cured by Liq. Arsenicalis

**Published:** 1811-06

**Authors:** W. H. Leadbeater

**Affiliations:** Overton, Hants


### *To the Editors of the Medical and Physical Journal.*

GENTLEMEN,

I BEG leave to hand you for insertion in your valuable Journal, the following case of periodical spasm entirely subdued by the use of the liquor arsenicalis; thinking it contains some facts, which may greatly tend to establish its more frequent, but at the same time discriminate use, in most disorders where there is a regular intermission.

I am, Gentlemen,

Yours, &c.

W. H. LEADBEATER.

*Overton, Hants, 2d April,* 1811.

### CASE.

On the 30th of December, 1810, I was called in to see a young lady, aged 18, whom, I was informed, had been labouring under a hard dry cough, for a month previous to the above date; she had a trifling pain in her side, bowels regular, pulse rather low, menstruates regularly.

Considering the case as nervous, I recommended a change of air, and at the same time, the following medicines. R. Mist. amygd. ℥ix. tinct. opii. gutt. viii. oxym. scillæ ℥i. M. ft. haustus ter in die sumendus.

 These

These medicines were continued with the best effect until the 5th of January following, when finding the cough entirely gone, and some degree of debility remaining, I ordered the following :—R. Decoct. cascarillæ ʒix. oxym. scillæ ʒi. vin. ipecac. gutt. v. tinc. cardam. C. ʒss. M. ft. haust. sumat. unum ter in die.

This plan pursued for a fortnight had the effect of bringing my patient into, apparently, perfect health. On the 20th of the same month, I was sent for in immediate haste to revisit her; I found her on a bed labouring under a most violent spasmodic affection of nearly the whole muscular system; the spasm was so violent as to require two persons to hold her in order to prevent her throwing herself off the bed. I immediately ordered her the following mixture :- R. Tinc. valer. ammon. ʒiij. opii. gutt. lx. mist. moschi. ℥vss. M. ft. mistura capt. coch. iij. ampl. 3tia. quaque hora.

In about ten hours the spasms had in a great measure subsided; on inquiry I found it to be four or five days previous to the time of her expecting the menses. I ordered her into the warm bath, and left her for the evening, desiring she might take a cup of strong coffee occasionally, and to continue her medicine for the night. The following morning I visited her and found her free from spasm, but ordered her the warm bath again, in order to accelerate the menses, this however, had not the desired effect. On the 24th instant she was again attacked with spasm, affecting the larynx only, but this in a most violent degree, so much so as to alarm every bye-stander; it did not put on the appearance of globulus hystericus, but exactly resembled a person in the last stage of suffocation. During the whole time, eight hours, she was frequently able to swallow barley water, which seemed to give her momentary relief. Ordered—R. Hydrarg. submuriat. gr. ss. ext. hyoscyam. gr. j. ext. conii. gr. iv. M. ft. Bolum. sumat unum 4ta. quaque hora cum haust. sequent. R. Ammoniæ carbonat. gr. v. conf. amygd. ʒj. mist. camphor. ʒix. spt. æther. C. ʒss. M. ft. haustus.

After twice taking the above medicines the spasm considerably abated, but was not entirely removed. The warm bath was continued every evening, and the above medicines taken three times a day, until the 27th instant, when the menses appeared, which had the effect of removing every unpleasant symptom, but at the same time leaving a great degree of debility. On the 3d of February the menses having subsided, I again put her on the tonic plan, with the cascarilla draughts as before, with the addition of the pills, as follows :—R. Pilul. ferri cum myrrh. pilul. galban. C. sing. ʒj. M. ft. Pilul. xx. sumat. iij. ter in die cum haust. cascarilla.

On the 10th of February, the day fortnight, from the last period of menstruation, she was again attacked as before, with the addition of a most violent cough, resembling, as nearly as possible, the barking of a dog, every two or three minutes; this, as before, was relieved by a free use of laudanum. On the 13th instant, exactly at the same hour, she had a return of the spasm as before; this continued eight hours and then went off. It now first struck me if we could gain a regular intermission, the liquor arsenicalis might be of great service; however, I determined on seeing the case more fully established, previous to ordering it; on the 15th the spasm returned at the same hour, was more violent and of longer duration. On the 16th, 17th, and 18th, it returned regularly at the end of the twenty-four hours. The case being now so distinctly periodical, I ordered the following—R. Pulv. trag. comp. gr. v. aq. distillat. ʒix. spt. cinnam. ʒj. liq. arsenic. gutt. v. M. ft. haustus sumat unum ter in die, augendo dosin liq. arsenic. gradatim ad guttas decem.

On the 20th the spasm returned much as usual; on the 21st it was less violent, and much shorter; the medicines were continued until the 29th, when the spasm had entirely left her; she has since menstruated without any inconvenience, and is now in perfect health. I recommended change of air through the whole, but her friends would not accede to it, under the idea that she was not able to undertake a journey.

I have, within the last six months, had several cases of periodical headachs, which have always given way to the use of the liquor arsenicalis; my professional engagements have prevented my keeping an account of these cases.

